# Association between smoking and the clinical status of adult patients with moderate-severe atopic dermatitis in the China Atopic Dermatitis Registry (ChinaSTAD): a cross-sectional study

**DOI:** 10.3389/fmed.2026.1849528

**Published:** 2026-08-31

**Authors:** Yingzhe Yu, Lu Cao, Xin Fan, Lingyi Lu, Sihan Wang, Jianzhong Zhang, Bingjiang Lin

**Affiliations:** 1 Department of Dermatology, The First Affiliated Hospital of Ningbo University, Ningbo, China; 2 Department of Dermatology, Peking University People’s Hospital, Beijing, China

**Keywords:** atopic dermatitis, Chinese, cross-sectional study, moderate to severe, smoking

## Abstract

**Background:**

Atopic dermatitis (AD) is a common inflammatory skin disease influenced by multiple factors such as genetic, environmental and lifestyle factors. This study explored the association between smoking and AD.

**Methods:**

The China Atopic Dermatitis Registry (ChinaSTAD) is a nationwide observational registry of patients with moderate to severe AD in China. We conducted a cross-sectional analysis of adult patients enrolled from July 2021 to April 2023 across 26 provinces. Patients were categorized as ever-smokers or non-smokers based on smoking history. Propensity scores were estimated using demographic and socioeconomic covariates, with 1:1 nearest neighbor matching applied. Associations between smoking status and clinical/patient-reported outcomes were evaluated using linear regression models with robust standard errors. Sensitivity analyses were performed by comparing results from unmatched, matched and doubly robust approaches.

**Results:**

After propensity scores matching, baseline characteristics were well-balanced. In the doubly robust analyses, smoking was associated with greater body surface area (BSA) values (adjusted mean difference 2.29, 95% CI 0.22–4.36), and showed a weak association with Patient Oriented Eczema Measure (POEM) scores (adjusted mean difference 0.59, 95% CI −0.00 to 1.19). In contrast, no clear association was found between smoking and other patient-reported outcomes and quality of life indicators. Sensitivity analysis showed that effect estimates varied across strategies and were generally attenuated after doubly robust adjustment.

**Conclusion:**

In this study, smoking was associated with BSA, but with weak or no association with disease severity, clinical phenotype or quality of life scores. Further prospective studies are required to clarify the relationship between smoking and AD.

## Introduction

Atopic dermatitis (AD) is a chronic inflammatory skin disease, affecting both children and adults worldwide. Globally, approximately 101.27 million adults and 102.78 million children are suffering from AD, with estimated prevalence of 1.95% (1.37%–2.61%) and 3.96% (2.81%–5.27%), respectively (1). The prevalence of AD also varies across geographic regions and countries, presenting diverse clinical phenotypes worldwide (2). Moreover, disease manifestations of AD were also distinct depending on different stages, ranging from exudation, papules, and plaques with itching in the acute phase to mossy lesions and hypertrophy in the chronic phase (3). In addition to the cutaneous manifestations, AD is associated with multiple comorbidities, including allergic rhinitis, rhino-conjunctivitis, asthma, and secondary infections (3). Besides, AD harms sufferers’ mentality. Population-based data from Korean have demonstrated a higher risk of suicidal thoughts among individuals with AD (4). Consistently, the Global Burden of Disease study also showed AD had the highest disease burden among skin diseases measured by disability-adjusted life-years (3, 5).

The pathogenesis of AD has not yet been fully elucidated. However, a range of predisposing factors (including genetic, immune dysregulation, environmental factors, skin barrier dysfunction, and microbial dysbiosis) as well as trigger factors have been proven to play a role in its development (3). Nowadays, an increasing body of studies has explored the relationship between lifestyle factors and AD. A sedentary lifestyle, physical activities, alcoholic consumption, family background, and even quality of diet have been reported to be significantly associated with AD presentation and severity according to the research conducted in Singapore and Malaysia (6). An international web-based survey indicated a higher prevalence of AD among smokers and heavy drinkers compared with non-smokers and non-drinkers across multiple countries (7). Meanwhile, smoking, including former smoking, has also been associated with severe AD (8). Correspondingly, study from Germany, has shown that AD patients have a higher proportion of addiction to alcohol consumption, drug use, and Internet compared with the general population (9).

To date, comprehensive research examining the impact of cigarette smoking on the clinical phenotype and severity of AD remains scarce. Furthermore, more existing studies were conducted in Europe, whereas data from Asian populations are limited. Therefore, the present study aimed to further investigate the association between smoking status and the clinical characteristics of AD among Chinese adult patients with moderate-to-severe disease.

## Materials and methods

### Patient and methods

The ChinaSTAD study is an ongoing nationwide registry in China (ClinicalTrials.govidentifier: NCT05023668) for patients diagnosed with moderate-to-severe AD (SCORAD score ≥ 25) and aged ≥ 12 years old. The study was approved by the Ethics Committee of the Peking University People’s Hospital (No. 2021PHB076-001; 7 April 2021) and by the local ethics committees of participating centers. The study was conducted in accordance with the Declaration of Helsinki. And the written informed consents were obtained from all enrolled participants.

For this analysis, data were collected from the baseline visit of enrolled patients between July 2021 and April 2023 from 81 hospitals across 26 provinces in China. After excluding minors and missing values, a total of 4,337 enrolment records were included for further analysis. The patients were categorized into two groups: ever-smokers and non-smokers according to smoke status. Ever smokers included former and current smokers, reflecting any history of cigarette use. Participants reported information on their date of birth, sex, marital status, level of education, occupation type, weight, height, ethnicity, and residence. Correspondingly, physicians obtained the clinical data including Body Surface Area (BSA), Scoring of Atopic Dermatitis (SCORAD), Patient Oriented Eczema Measure (POEM), Atopic Dermatitis Control Tool (ADCT), Dermatology Life Quality Index (DLQI), and Numerical Rating Scale (NRS). To further explore the characteristics of AD, we used the SCORAD score for intensity and subjective symptoms to compare different clinical manifestations between the two groups in the following six aspects: erythema, oedema/papules, oozing/crusts, excoriation, lichenification, and dryness.

### Statistical analysis

Continuous variables are presented as mean ± standard deviation (SD), and categorical variable as number (percentage). Baseline characteristics were compared between groups of ever-smokers and non-smokers, and covariate balance was assessed by standardized mean differences (SMD), with an absolute SMD < 0.10 indicating adequate balance. To reduce confounding factors, propensity score matching (PSM) was used to create comparable groups. Propensity scores were estimated using a logistic regression model including age, sex, body mass index (BMI), marital status, education level, occupation, and residence. One-to-one nearest-neighbor matching (without replacement) was performed on the logit values of the propensity score using calipers with a width of 0.2 standard deviations. After matching, covariate balance was reassessed using SMD and visualized with Love plots.

All primary analyses were conducted in the propensity score matched cohort. Descriptive statistics were performed on the outcome measures in both groups. Linear regression models fitted in the matched sample were used to examine the associations between smoking status and continuous outcome measures (including SCORAD, POEM, DLQI, ADCT, BSA, NRS and symptom subscores). Robust standard errors were used to account for matched pairs, and results were expressed as adjusted mean differences with 95% confidence intervals (CI). To adjust for residual imbalance in covariates, regression adjustments were further made for age, occupation, and education level in the matched samples to construct a doubly robust estimation model. The adjusted mean differences, 95% confidence intervals (95%CI), and *P*-values were derived from this model. Furthermore, sensitivity analyses were conducted to compare the results of multivariate linear regression in the unmatched samples, the matched samples, and the doubly robust models in the matched samples. The robustness of the results was evaluated by comparing the consistency of the direction and magnitude of the effect under different methods.

All statistical test were two-sided and statistical analyses were performed using R software (version4.3.1).

## Results

### Patient characteristics

**FIGURE 1:**
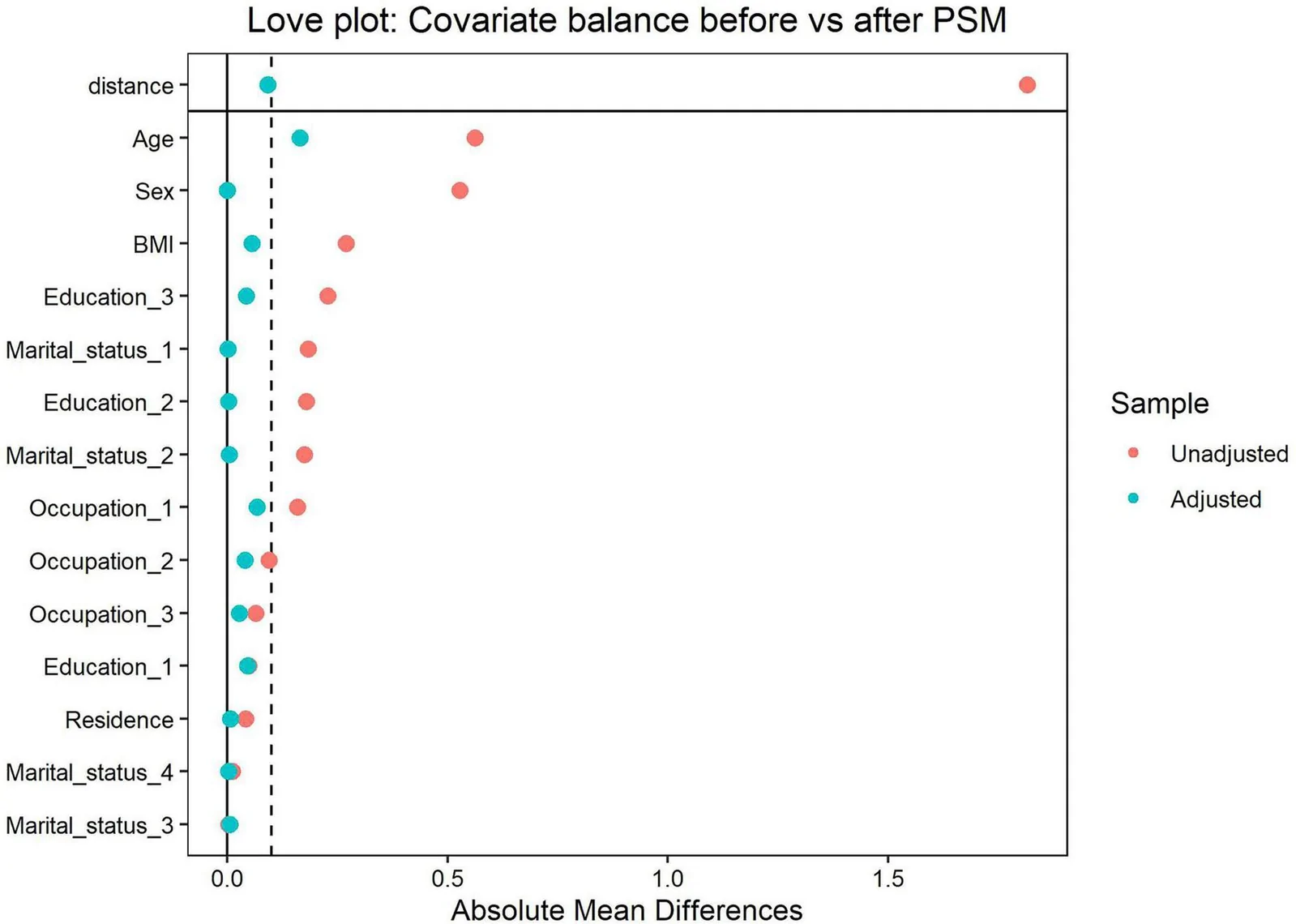
Study flow chart. A total of 5,225 patients were initially included. Individuals with missing data or scores, those whose disease severity did not meet the criteria, and minors were excluded. Subsequently, patients were divided into different subgroups according to smoking status, and propensity score mating was applied.

A total of 5,225 patients were enrolled, of whom 4,337 participants met inclusion criteria (3,098 non-smokers and 1,239 ever-smokers). One to one propensity score matching yielded 1,017 matched pairs (2,034 patients), which constituted the final analytic cohort (Figure 1).

**FIGURE 2:**
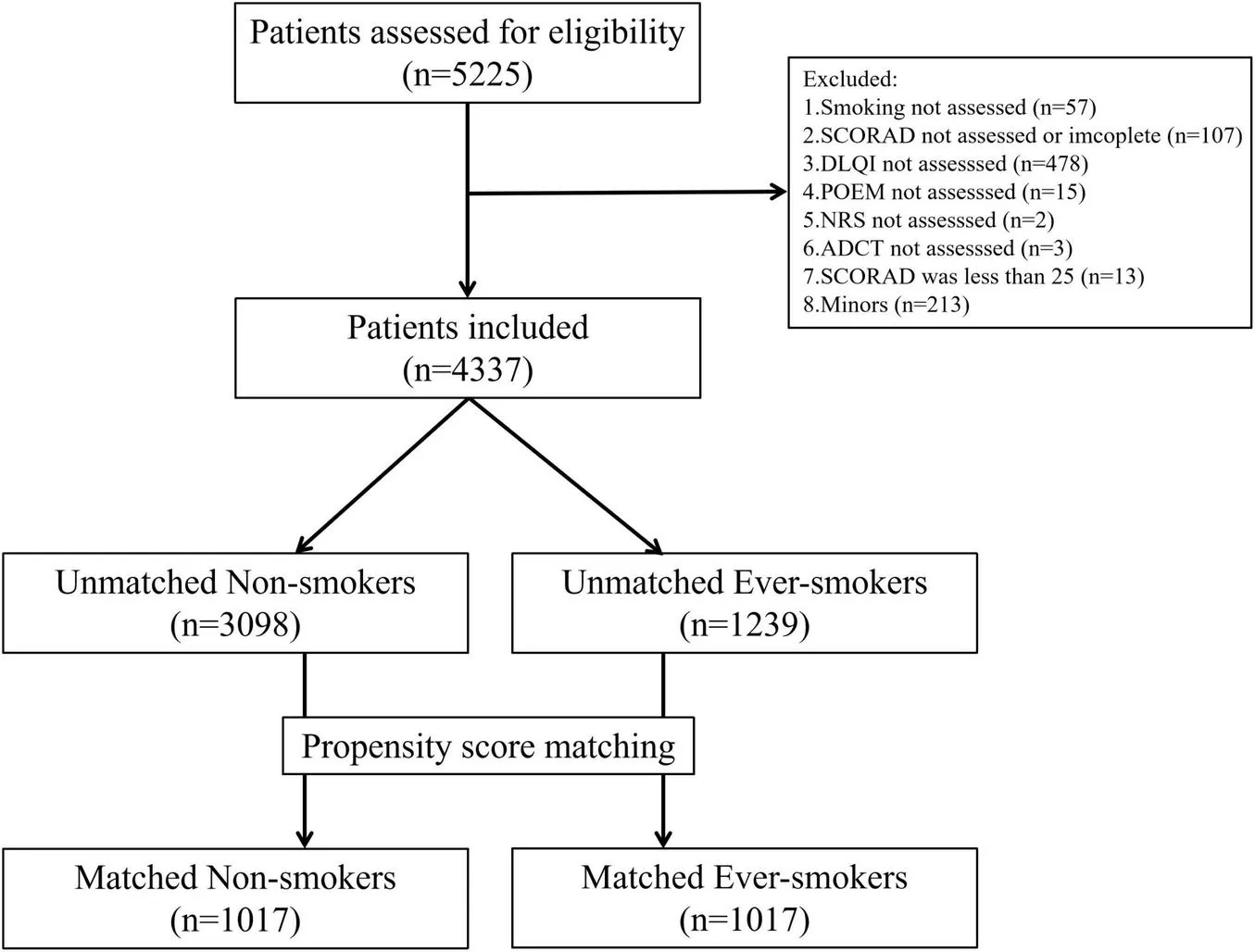
Love spot of covariate balance before and after propensity score matching. Standardized mean differences (SMD) of baseline covariates between non-smokers and ever-smokers before and after propensity score matching are displayed. Orange circles represent the unmatched sample, and green circles represent the matched sample. The vertical dashed line indicates an absolute SMD of 0.10, which was considered the threshold for adequate balance.

Before matching, ever-smokers and non-smokers differed substantially in age, sex, BMI, marital status, education, occupation, and residence, with several SMD exceeding 0.10, indicating notable baseline imbalance. After propensity score matching, covariate balance improved markedly, suggesting adequate balance between the groups (Figure 2 and Table 1). Ethnicity did not differ significantly between the groups in the unmatched cohort (SMD = 0.020) and was therefore not included in the propensity score model.

**TABLE 1:** General patient characteristics before and after propensity score matching.

Characteristic	Unmatched non-smokers (*n* = 3,098)	Unmatched ever-smokers (*n* = 1,239)	SMD	Matched non-smokers (*n* = 1,017)	Matched ever-smokers (*n* = 1,017)	SMD
Age (years), mean ± SD	42.58 ± 19.07	52.77 ± 18.13	0.548	49.41 ± 19.59	52.40 ± 19.30	0.154
Sex *N* (%)			1.368			<0.001
Male	1280 (41.3)	1166 (94.1)		944 (92.8)	944 (92.8)	–
Female	1818 (58.7)	73 (5.9)		73 (7.2)	73 (7.2)	–
BMI, mean ± SD	22.79 ± 3.52	23.68 ± 3.26	0.260	23.80 ± 3.31	23.61 ± 3.33	0.055
Marital status *N* (%)			0.444			0.058
Unmarried	1,002 (32.3)	184 (14.9)		188 (18.5)	184 (18.1)	–
Married	2,025 (65.4)	1,037 (83.7)		815 (80.1)	816 (80.2)	–
Divorced	25 (0.8)	13 (1.0)		7 (0.7)	12 (1.2)	–
Widowed	46 (1.5)	5 (0.4)		7 (0.7)	5 (0.5)	–
Education *N* (%)			0.468			0.144
College and above	1,918 (61.9)	485 (39.1)		525 (51.6)	481 (47.3)	–
Middle school	891 (28.8)	579 (46.7)		386 (38.0)	383 (37.7)	–
Primary and below	289 (9.3)	175 (14.1)		106 (10.4)	153 (15.0)	–
Occupation *N* (%)			0.353			0.144
Mainly manual work	358 (11.6)	260 (21.0)		488 (48.0)	419 (41.2)	–
Mainly mental work	1,668 (53.8)	470 (37.9)		183 (18.0)	224 (22.0)	–
Retired or unemployed	1,072 (34.6)	509 (41.1)		346 (34.0)	374 (36.8)	–
Ethnicity *N* (%)			0.020			0.042
Han Chinese	2,992 (96.6)	1,201 (96.9)		992 (97.5)	985 (96.9)	–
Others	106 (3.4)	38 (3.1)		25 (2.5)	32 (3.1)	–
Residence *N* (%)			0.131			0.021
City	2,803 (90.5)	1,069 (86.3)		898 (88.3)	891 (87.6)	–
Countryside	295 (9.5)	170 (13.7)		119 (11.7)	126 (12.4)	–

Values are presented as mean ± SD or *n* (%). Propensity scores were estimated using age, sex, BMI, marital status, education, occupation, and residence. Standardized mean differences were used to assess covariate balance, with values <0.10 indicating acceptable balance. Ever smokers included both current and former smokers.

### Severity of atopic dermatitis

In the matched cohort, ever-smokers tended to have slightly higher mean scores for BSA, SCORAD, POEM, ADCT, DLQI, and NRS compared with non-smokers. However, the absolute differences were small (Table 2).

**TABLE 2:** Associations between smoking and patient-reported and composite outcomes in the matched cohort.

Outcome	Ever-smokers, mean ± SD	Non-smokers, mean ± SD	Adjusted mean difference (95% CI)	*P*-value
BSA (%)	35.66 ± 24.52	32.71 ± 23.52	2.29 (0.22, 4.36)	0.030*
SCORAD	57.41 ± 16.90	55.47 ± 16.52	1.40 (−0.03, 2.82)	0.055
POEM	16.46 ± 7.09	15.80 ± 6.70	0.59 (−0.00, 1.19)	0.050
ADCT	14.27 ± 5.65	13.66 ± 5.63	0.48 (−0.01, 0.98)	0.056
DLQI	11.36 ± 7.49	11.02 ± 6.87	0.46 (−0.17, 1.09)	0.151
NRS	7.10 ± 2.38	7.04 ± 2.30	−0.02 (−0.22, 0.19)	0.878

Adjusted mean differences and 95% confidence intervals were estimated using linear regression models accounting for matched pairs with cluster-robust standard errors. *p < 0.05.

In doubly robust models fitted in the matched sample, significantly higher BSA was observed in the ever-smokers (adjusted mean difference 2.29, 95% CI 0.22–4.36). And smoking showed a weak and borderline significant association with higher POEM scores (adjusted mean difference 0.59, 95% CI −0.00 to 1.19). No significant differences were observed for SCORAD, ADCT, DLQI, and NRS, with confidence intervals spanning the null.

### Clinical manifestations of atopic dermatitis

Associations between smoking and clinical manifestation are shown in Table 3. The ever-smokers tended to have slightly higher scores for oedema/papules, whereas no marked differences were observed for erythema, oozing/crusts, excoriation, lichenification, or dryness.

**TABLE 3:** Associations between smoking individual sign scores in the matched cohort.

Outcome	Ever-smokers, mean ± SD	Non-smokers, mean ± SD	Adjusted mean difference (95% CI)	*P*-value
Erythema	2.11 ± 0.72	2.08 ± 0.72	0.03 (−0.04, 0.09)	0.447
Oedema/papules	1.71 ± 0.85	1.62 ± 0.87	0.09 (0.02, 0.16)	0.017*
Oozing/crusts	1.57 ± 0.93	1.50 ± 0.90	0.07 (−0.01, 0.15)	0.101
Excoriation	1.80 ± 0.83	1.75 ± 0.83	0.03 (−0.04, 0.10)	0.395
Lichenification	1.67 ± 0.91	1.63 ± 0.91	0.03 (−0.05, 0.11)	0.493
Dryness	2.06 ± 0.80	2.05 ± 0.78	−0.00 (−0.07, 0.07)	0.989

Effect estimates represent adjusted mean differences between ever-smokers and non-smokers derived from linear regression models with doubly robust analyses. *p < 0.05.

In the doubly robust model, smoking was weakly associated with oedema/papules (adjusted mean difference 0.090, 95% CI 0.016–0.164), while no significant associations were found for other clinical manifestations.

### Sensitivity analysis for models

Sensitivity analyses are presented in Table 4. Effect estimates varied across unmatched multivariable regression, propensity score matching analyses, and doubly robust models, indicating sensitivity to the extent of confounding control. Across analytic approaches, the association with BSA remained directionally consistent (95% CIs did not included the null). Meantime, in the unmatched model, smoking was significantly associated with higher POEM scores (adjusted mean difference 0.70, 95% CI 0.17–1.23). And the association remained consistent in the propensity score-matched model (adjusted mean difference 0.66, 95% CI 0.07–1.25). However, after applying the doubly robust analyses to further account for residual confounding, the effect size was attenuated to borderline significance (adjusted mean difference 0.59, 95% CI −0.00 to 1.19). In contrast, estimates for SCORAD, ADCT, DLQI, and NRS were attenuated and imprecise with confidence intervals crossing the null, indicating no clear association after confounding control.

**TABLE 4:** Comparison of results across analytic approaches.

Outcome	Unmatched	Unmatched (p)	PSM_only	PSM_only (p)	PSM_DR	PSM_DR (p)
BSA (%)	1.70 (−0.11, 3.51)	0.065	2.95 (0.87, 5.02)	0.005	2.29 (0.22, 4.36)	0.030
SCORAD	1.39 (0.15, 2.64)	0.029	1.95 (0.52, 3.38)	0.008	1.40 (−0.03, 2.82)	0.055
POEM	0.70 (0.17, 1.23)	0.010	0.66 (0.07, 1.25)	0.028	0.59 (−0.00, 1.19)	0.050
ADCT	0.68 (0.25, 1.11)	0.002	0.61 (0.12, 1.10)	0.014	0.48 (−0.01, 0.98)	0.056
DLQI	0.55 (−0.00, 1.10)	0.052	0.34 (−0.29, 0.96)	0.289	0.46 (−0.17, 1.09)	0.151
NRS	0.10 (−0.08, 0.28)	0.255	0.06 (−0.14, 0.26)	0.564	−0.02 (−0.22, 0.19)	0.878

Unmatched adjusted models were fitted using multivariable linear regression. PSM_only estimates were derived from regression models accounting for matched pairs. PSM_DR refered to the models that doubly robust estimation used to further adjust the residual imbalances. Values represented as adjusted mean differences (95% confidence intervals).

## Discussion

Smoking is a traditional public health problem that has long been identified as a risk factor for multiple diseases, including malignancies and chronic-inflammatory diseases (10, 11). The present study explored the association between cigarette use and disease manifestations and clinical severity of AD in the Chinese population using data from the China Atopic Dermatitis Registry (ChinaSTAD) Study.

Align with previous studies, our findings showed that the population of smokers is predominantly male, generally older, and less educated (12). Meanwhile, smokers in this study were more likely to be married, currently retired or unemployed, and at a higher risk of obesity compared to non-smokers.

Subsequently, we examined the relationship between smoking status and clinical outcomes of AD using propensity score matching and doubly robust analyses. After balancing baseline characteristics between ever-smokers and non-smokers, smoking remained weakly associated with greater BSA involvement and was associated with slightly higher POEM scores and more pronounced edema/papulation. In contrast, associations with other patient-reported outcomes and health-related quality-of-life measures were inconsistent, with most confidence intervals crossing the null value. Overall, these findings suggest that smoking is associated with certain aspects of disease expression rather than being a major determinant of overall disease severity. Although the observed effect were modest and should not be overinterpreted as clinically substantial, even small differences in BSA may still be relevant in clinical practice because lesion extent contributes to treatment selection, disease monitoring, and assessment of therapeutic response. Therefore, our findings support a cautious interpretation that smoking may exert a limited but measurable influence on objective disease extent, while its impact on broader clinical outcomes appears relatively small after rigorous adjustment for confounding.

Prior epidemiological evidence on smoking and AD severity has been mixed. Some studies showed that smoking is associated with the incidence of AD and is a risk factor for its development regardless of sex or age (13–16). A systematic review of observational studies suggested that both active and passive smoking may be associated with AD, but highlighted substantial heterogeneity across populations and study designs (15). Data from the TREATgermany registry indicated that smoking patients may exhibit a higher burden and distinct lesion distribution patterns, particularly in adult-onset AD (17). A population-based study of the Lifelines Cohort Study have also linked heavier smoking exposure (e.g., higher pack-year history) with an increased likelihood of moderate-to-severe AD, suggesting a potential dose-related association (18). Several studies have explored the pro-inflammatory mechanisms of tobacco. Smokers exhibited elevated serum IgE levels as smoking seems to facilitate the development of an atopic predisposition and enhance individual susceptibility to allergens (19, 20). Additionally, chronic skin inflammation such as atopic dermatitis is associated with excessive production of reactive oxygen species, which is known as oxidative stress and eventually exceeds the defenses of the antioxidant system (21). Cigarette contains high level of reactive oxygen species and reactive nitrogen species, which are the root cause of oxidative stress (22, 23). Notably, however, a substantial body of epidemiological evidence has reported no clear link between smoking and AD. For example, a cross-sectional study in Oslo found no association between smoking and reported AD or hand eczema (24). Similarly, analyses from the Korean National Health and Nutrition Examination Survey showed no significant association between smoking status and adult AD prevalence (25). Earlier case-control studies also reported that smoking was not a significant risk factor for AD, reinforcing the lack of a consistent association across different settings (26). In line with these literatures, our findings suggest that smoking may be weakly associated with objective severity (BSA), but the impact on patient-reported outcomes and quality of life is imprecise after rigorous confounding control, underscoring the need for cautious interpretation in cross-sectional real-world data.

The differences in effect estimates under different outcomes and analytical strategies highlight the importance of controlling for confounding factors in real-world data. The baseline differences were significant, while the correlation weakened after matching and further adjustment, suggesting that some of the crude associations were driven by demographic and socioeconomic factors. Moreover, patient-reported outcomes and quality of life indicators were influenced by various non-disease factors, such as the psychosocial environment and access to healthcare services, which could further diminish any direct association with smoking status.

It should be noted that this study has several limitations. First, the cross-sectional study design does not allow for inferring time order or causality, nor can it rule out the possibility of inverse associations. Second, smoking exposure was defined dichotomously, lacking information such as dose, duration, or time to cessation, which limits dose-response assessment. The use of the “ever-smokers” definition may have introduced exposure misclassification, potentially attenuating true associations. Future studies incorporating smoking intensity, duration, pack-years, current versus former smoking status, and smoking cessation history are warranted to clarify potential dose-response relationships. Third, despite the use of propensity score matching and a dual robust model, residual confounding cannot be completely excluded. Some potentially relevant factors, such as passive smoking exposure and environmental exposures, were not systematically collected in the registry. In addition, several available variables, including treatment history, and selected comorbidities, contained substantial missing data. Including these variables in the propensity score model would have markedly reduced the analyzable sample size and potentially introduced selection bias. Consequently, only covariates with high completeness and established clinical relevance were included in the primary analyses. Future registry studies with more comprehensive and complete data collection are warranted to further minimize residual confounding. For instance, previous studies have found the impact of passive smoking on the severity of AD, but in this study, the confounding factor of passive smoking could not be avoided, which may affect the persuasiveness of the results to some extent (27). Finally, measurement errors and missing data may have contributed to the inaccuracies in some results.

## Conclusion

This study aimed to explore the association between smoking status and clinical outcomes in Chinese patients with moderate-to severe AD. We found that smoking status was associated with greater lesion extent, but no significant differences were found in rash morphology and severity. Further comprehensive, large-scale prospective studies are needed to fully explore the correlation.

## Data Availability

The datasets analyzed during the current study are not publicly available but are available from the corresponding author upon reasonable request. Requests to access the datasets should be directed to Jianzhong Zhang at rmzjz@126.com.
